# Supplementary material for: Effects of Ovine Monocyte-Derived Macrophage Infection by Recently Isolated Toxoplasma gondii Strains Showing Different Phenotypic Traits
Source: Animals (Basel). 2022 Dec 7;12(24):3453. doi: 10.3390/ani12243453 (PMC9774764; doi:10.3390/ani12243453)
Supplement: Supplementary file 1 [file animals-12-03453-s001.zip › animals-2006104-supplementary.pdf]

Table S1. Sequences of primers used for quantitative real-time PCR (qPCR) and standard curve data.

Target[1]	Primer	Primer sequence (5´-3´)	Product size (bp)	R^2	Slope
β -actin[a] (NM_001009784.1)	β -actin-UP	ACACCGCAACCAGTTCGCCAT	216	0.995	-3.334
	β -actin-RP	GTCAGGATGCCTCTCTTGCT			
IL-4 [a] (XM_004008636.1)	IL4-UP	CTGCCCCAAAGAACGCAACT	154	0.996	-3.659
	IL4-RP	TCATTCACAGAACAGGTCTTGCTT			
IL-10 [a] (NM_001009327.1)	IL10-UP	TGCTGGATGACTTTAAGGGTTACC	60	0.999	-3.957
	IL10-RP	AAAACTGGATCATTTCCGACAAG			
IL-12p40 [a] (NM_001009438.1)	IL12-UP	ATGGAATTTGGTCCACTGATATT	95	0.995	-3.613
	IL12-RP	GTGAAGTGTCCAGAATAATCCTTT			
TNF-α [a] (NM_001024860.1)	TNF-α-UP	CCAGAGGGAAGAGCAGTCC	126	0.944	-3.131
	TNF-α-RP	GGAGCGCTGATGTTGGCTAC			
TGF-β [a] (NM_001009400.1)	TGF-β-UP	GGTGGAATACGGCAACAAAA	117	0.989	-3.556
	TGF-β-RP	CGAGAGAGCAACACAGGTTC			
IL17 [b] (XM_004018887.4)	IL-17UP	AGTCTGGTGGCTCTTGTGAA	113	0.991	-3.641
	IL-17RP	TTAACGATGTTCAGGTTGAC			
IL-6 [b] (X68723.1)	IL-6-UP	CTGGGTTCAATCAGGCGATT	150	0.987	-3.742
	IL-6-RP	GGATCTGGATCAGTGTTCTGA			
IL1-α [c] (XM_012159802.3)	IL1-α-UP	CTGGGAAAAGCATGGGTCTA	89	0.983	-3.317
	IL1-α-RP	TGTGCACCAGTTTTTCTTGC			
iNOS[b] (AF223942.1)	iNOS-UP	GAGGAAGTGGGCAGAAGGAT	176	0.999	-3.34
	iNOS-RP	AAGTACATGCAGAACGAGTA			

[1] NCBI accesion number are for ovine cDNA sequences. Primer annealing was also checked with the *Ovis aries* genomic DNA sequences [a] Primers described by Arranz et al., 2016. [b] Primers described by Arteche-Villasol et al., 2021. [c]Primers first described in this study.

Table S2. Mean and standard deviation of the cell infection rate (cIR) according to each isolate used for infection, the number of experiment and the number of replicates made of each isolate.

Isolate (genotype/origin)	Experiment no	Replica	Cell infection rate		Total
TgShSp1 (#3/abortion)	1	*A*	70.29 ± 9.01	60.94 ±12.62	60.16 ± 12.71
		B	51.60 ± 16.32		
	2	*A*	65.21 ± 13.01	60.67 ±10.01	
		B	56.14 ± 7.02		
	3	*A*	67.47 ± 23.01	58.88 ±15.51	
		B	50.30 ± 8.01		
TgShSp2 (#1/abortion)	1	*A*	87.12 ± 13.02	81.24± 12.12	80.15 ± 8.91
		B	75.37 ± 11.23		
	2	*A*	85.46 ± 6.04	80.26 ± 6.53	
		B	75.06 ± 7.02		
	3	*A*	81.90 ± 7.01	78.97 ± 8.07	
		B	76.05 ± 9.05		
TgShSp3 (#3/abortion)	1	*A*	75.50 ± 7.02	74.71 ± 7.51	64.92 ± 9.85
		B	73.93 ± 8.01		
	2	*A*	59.00 ± 14.03	49.70 ±11.02	
		B	40.41 ± 7.04		
	3	*A*	75.40 ± 9.04	70.37 ±11.03	
		B	65.35 ± 13.02		
TgShSp11 (#3/myocardium of chronic infected sheep)	1	*A*	85.38 ± 11.03	81.18 ± 9.17	71.19 ± 9.13
		B	76.98 ± 7.32		
	2	*A*	63.34 ± 10.34	69.77 ± 9.22	
		B	76.21 ± 8.11		
	3	*A*	66.18 ± 7.01	62.62 ± 9.01	
		B	59.06 ± 11.01		
TgShSp16 (#3/myocardium of chronic infected sheep)	1	*A*	65.43 ± 20.02	70.44 ±19.02	71.98 ± 12.20
		B	75.46 ± 18.02		
	2	*A*	72.43 ± 14.02	71.18 ±10.03	
		B	69.93 ± 6.04		
	3	*A*	69.63 ± 7.02	74.34 ± 7.56	
		B	79.06 ± 8.11		
TgShSp24 (#2/myocardium of chronic infected sheep)	1	*A*	92.46 ± 5.03	93.21 ± 3.52	93.51 ± 5.71
		B	93.97 ± 2.02		
	2	*A*	89.66 ± 7.09	91.60 ± 6.06	
		B	93.55 ± 5.03		
	3	*A*	94.75 ± 12.00	95.73 ± 7.52	
		B	96.72 ± 3.04		

Table S3. Mean and standard deviation of the cell infection rate (McR) according to each isolate of infection used, the number of experiment and the number of replicates made of each isolate.

Isolate (genotype/origin)	Experiment no	Replica	Multiinfected cell rate		Total
TgShSp1 (#3/abortion)	1	*A*	58.97 ± 14.02	50.42 ± 14.06	40.95 ± 12.69
		B	41.87 ± 14.10		
	2	*A*	32.63 ± 10.00	33.78 ± 9.51	
		B	34.94 ± 9.02		
	3	*A*	49.28 ± 18.02	38.67 ± 14.52	
		B	28.07 ± 11.02		
TgShSp2 (#1/abortion)	1	*A*	83.27 ± 17.12	68.79 ± 13.06	56.21 ± 10.57
		B	54.31 ± 9.01		
	2	*A*	62.98 ±10.01	53.03 ± 8.55	
		B	43.08 ± 7.10		
	3	*A*	62.05 ± 7.10	46.81 ± 10.10	
		B	31.58 ± 13.10		
TgShSp3 (#3/abortion)	1	*A*	59.64 ± 16.01	55.43 ± 14.01	49.11 ±17.19
		B	51.23 ± 12.01		
	2	*A*	32.66 ± 17.01	38.47 ± 21.56	
		B	44.28 ± 26.12		
	3	*A*	59.31 ± 18.01	53.44 ± 16.01	
		B	47.58 ± 14.02		
TgShSp11 (#3/myocardium of chronic infected sheep)	1	*A*	83.92 ± 5.01	61.58 ± 4.01	44.84 ± 5.02
		B	39.25 ± 3.02		
	2	*A*	42.70 ± 7.01	38.15 ± 6.06	
		B	33.61 ± 5.12		
	3	*A*	51.16 ± 5.00	34.81 ± 5.00	
		B	18.46 ± 5.00		
TgShSp16 (#3/myocardium of chronic infected sheep)	1	*A*	59.91 ± 17.21	48.28 ± 13.11	47.76 ± 9.39
		B	36.66 ± 9.02		
	2	*A*	56.34 ± 16.12	41.02 ± 9.56	
		B	25.71 ± 3.00		
	3	*A*	70.77 ± 8.01	53.98 ± 5.51	
		B	37.20 ± 3.02		
TgShSp24 (#2/myocardium of chronic infected sheep)	1	*A*	81.28 ± 8.02	61.54 ± 5.56	65.03 ± 4.59
		B	41.80 ± 3.10		
	2	*A*	79.55 ± 10.00	60.56 ± 7.05	
		B	41.57 ± 4.11		
	3	*A*	98.19 ± 1.12	72.99 ± 1.17	
		B	47.79 ± 1.23		

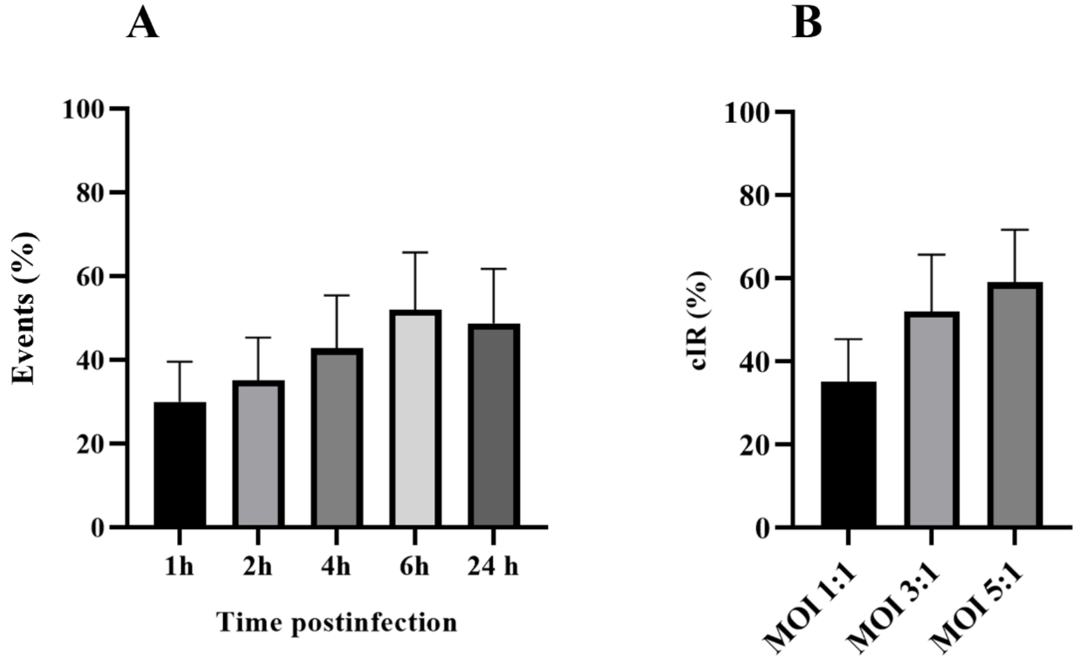

Figure S1. Previous in vitro studies carried out with *T. gondii* and ovine monocyte-derived macrophages to define time point of infection and MOI. **A.** Parasite invasion rates in ovine monocyte-derived macrophages infected by TgShSpain 1 isolate. Graphs represent percentage of internalized and intact tachyzoites studied at different time points. Each colum and error bar represents the mean and the SD. The total number of internalized tachyzoites was determined by single immunofluorescence staining of events followed by counting using an inverted fluorescence microscope. **B.** Graphs represent the cell infection rates at 6 hours postinfection as the percentage of infected cells and multi.infected cells in ovine monocyte.derived macrophages for TgShSpain1 isolate. Each column and error bar represents the mean value and SD using MOIs of 1, 3 and 5. The total number of cellsa and the number of infected cells was determined by double immunofluorescence staining followed by counting using and inverted fluorescence microscope.

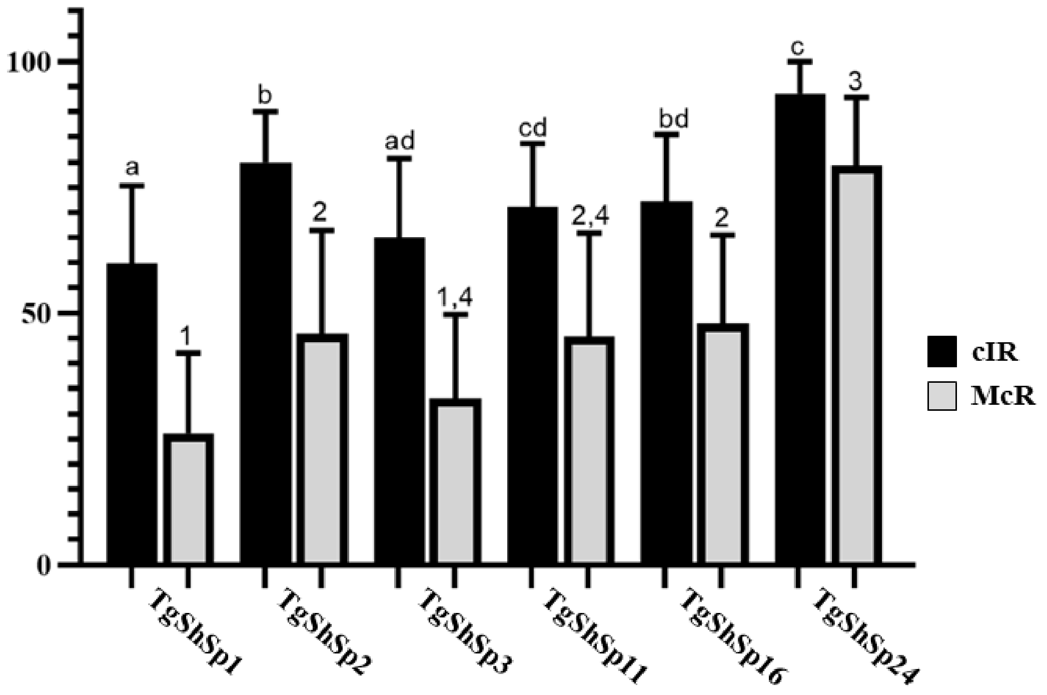

Figure S2. Cell infection rate (cIR) and multi-infected cell rate (McR) at 6 hpi in OvMØs for the six *Toxoplasma gondii* isolates using MOI of 3:1. The total number of cells, the number of infected cells (with intact tachyzoites) and the number of cells with multi-infection were determined by double immunofluorescence staining followed by counting using an inverted fluorescence microscope. Different superscript letters indicate statistically significant differences between pairs of the six isolates regarding cIR (Mann-Whitney test) ($p \leq 0.05$). Different superscript numbers indicate statistically significant differences between pairs of the six isolates regarding McR (Mann-Whitney test) ($p \leq 0.05$).

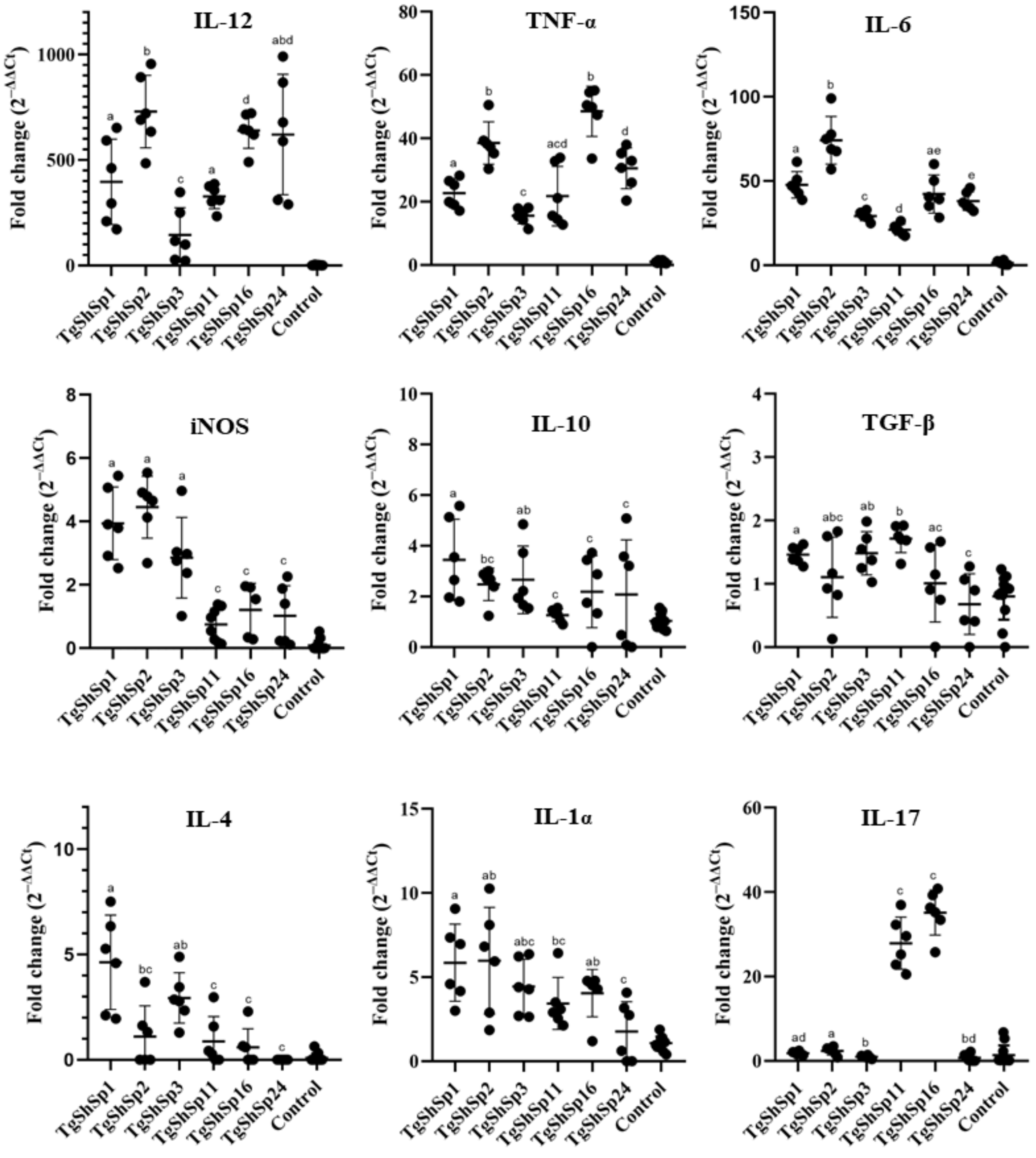

Figure S3. IL-12, TNF-α, IL-6, iNOS, IL-10, TGF-β, IL-4, IL-1α and IL-17 transcript expression. Scatter plot graphs of relative mRNA expression levels (as a fold change) of cytokines and iNOS in OvMØs infected with different *Toxoplasma gondii* isolates (MOI 3:1) at 6 hpi**.** Isolates: TgShSp1, TgShSp3, TgShSp11, TgShSp16, TgShSp2 and TgShSp24. Data represent mean and standard deviation. Different superscript letters indicate statistically significant differences between pairs of isolates (Mann-Whitney test) ($p \leq 0.05$).
